# 2025 Acknowledgment of *Journal of Virology Ad Hoc* Reviewers

**DOI:** 10.1128/jvi.01012-26

**Published:** 2026-08-18

**Authors:** Felicia Goodrum, Stacey Schultz-Cherry

**Affiliations:** 1 Geisel School of Medicine, Dartmouth College 3728, Hanover, New Hampshire, USA; 2 Host Microbe Interactions, St. Jude Children’s Research Hospital 5417, Memphis, Tennessee, USA

## EDITORIAL

On behalf of the editors of *Journal of Virology*, we acknowledge the following *ad hoc* reviewers for their time and effort in reviewing manuscripts for us during the past year. Your service is both invaluable and greatly appreciated. We would especially like to thank those who reviewed five or more papers last year (names in bold).

Karim Abdelkader

Elsayed M. Abdelwhab

Chantal Abergel

Hassanein Hassan Abozeid

Rachy Abraham

Jônatas S. Abrahão

Anas Haider Abu-Humaidan

Pakorn Aiewsakun

Hideki Aihara

Ramesh K. Akkina

Jonas Albarnaz

Pepe Alcami

Mia Madel Alfajaro

Irving Coy Allen

Andrew B. Allison

Covadonga Alonso

Sylvie Alonso

Sergio P. Alpuche-Lazcano

Nihal Altan-Bonnet

Jennifer Altomonte

Vorawit Ananphongmanee

Petronela Ancuta

Larry J. Anderson

Julien Andreani

Germán Andrés

Sarah A. Andrews

Laura Anfossi

Balakrishnan Anukumar

Hiroshi Aoki

Miguel A. Aranda

**Nancie M. Archin**

Daniel Ardisson-Araujo

Gerardo R. Argüello-Astorga

Minetaro Arita

Eurico Arruda

Vaithilingaraja Arumugaswami

Sassan Asgari

Felipe Lopes Assis

Doina Atanasiu

Thomas Aune

Teresa Aydillo

María A. Ayllón

Frank O. Aylward

Martin Fabian Bachmann

Cyril Badaut

Adam L. Bailey

Dalan Bailey

Goran Bajic

Steven F. Baker

Udeni B. Balasuriya

Thaisa Baldo

Megan Tierney Baldridge

Keir M. Balla

Arinjay Banerjee

Norbert Bannert

Benoit Barbeau

Stefanie Barbirz

Wendy S. Barclay

Michael D. Baron

Ian G. Barr

John T. Bates

Joshua Baty

Lisa Bauer

Fernando Bauermann

Sina Bavari

Daniel Becker

Martin Beer

Nadejda Beliakova-Bethell

Antje Beling

Gael Belliot

Sandrine Belouzard

Jessica Belser

Graham John Belsham

Robert B. Belshe

Eric Bergeron

Anthony Berglas

Ben Berkhout

Jacob Berrigan

Antonio Bertoletti

Sonja Best

Sachin Bhagchandani

Darshak Bhatt

David Bhella

Ashok Vishwambhar Bhosale

Stephane Biacchesi

Dennis Ken Bideshi

Craig J. Bierle

David M. Bisaro

Hugo Bisio

Paola Blanchette

Jorge Carlos Blanco

Rafael Blasco

Jesse D. Bloom

Guy Boivin

Jamie Bojko

Boris Bonaventure

Joseph Bondy-Denomy

Bryony C. Bonning

Lisa M. Bono

Alexander Borodavka

Eva Maria Borst

Alberto Bosque

Jens Bosse

Matteo Bosso

Leticia Botella

Eva Böttcher-Friebertshäuser

Evzen Boura

Chris Boutell

Andrew S. Bowman

David Boyd

Steven B. Bradfute

Curtis R. Brandt

Marta Santos Bravo

John Briggs

Mark A. Brimble

Matthijs C. Brouwer

Amanda Brown

Edward P. Browne

Emily A. Bruce

Zabrina L. Brumme

Astrid Bryon

Filemon Bucardo

Shilpa Buch

Julian Buchrieser

Javier Buesa

Remi Buisson

James Burke

Mark W. Burke

Alita R. Burmeister

Giacomo Butta

Jose Caaveiro

C. Joaquín Cáceres

Sarah Caddy

Valeria Cagno

J. Mauro Calabrese

Alexandre Rodrigues Calazans

Rafael K. Campos

Cláudio Wageck Canal

Bruno Canard

Thierry Candresse

Bin Cao

Jianhao Cao

**Yongchang Cao**

Yunlong Richard Cao

Adam Capoferri

Jeremy Carlton

George W. Carnell

Jillian Maree Carr

John Carr

Steven D. Carson

José M. Casasnovas

James Brett Case

John L. Casey

Marina Caskey

Guillaume Castel

Araceli G. Castillo

Ethel Cesarman

Inho Cha

Irwin M. Chaiken

Kuan Rong Chan

Pearl Chan

Ajit Chande

Hui-Wen Chang

Rudragouda Channappanavar

Shruti Chatterjee

Soma Chattopadhyay

Benjamin Chen

Chaoping Chen

Duanduan Chen

Jing Chen

**Jiong Chen**

Jueqi Chen

**Nanhua Chen**

Wei Chen

Wei-Chao Chen

Weiqiang Chen

Wentian Chen

Xi Chen

Yunyu Chen

Zhao-Shan Chen

Zhiwei Chen

Gong Cheng

Xiaofei Cheng

Ziqiang Cheng

Leonid V. Chernomordik

Joshua L. Cherry

Keng Yih Chew

John A. Chiorini

Elliott Chiu

Petr Chlanda

Kyoung-Oh Cho

Won Kyong Cho

**Kang-Seuk Choi**

Shafiqul I. Chowdhury

Bei-Bei Chu

Xia Chuai

Andrea Cimarelli

Kevin Ciminski

Alex E. Clark

Penny Clarke

Michael Clawson

Donald M. Coen

Éric A. Cohen

Charles Cole

Jackie L. Collier

Che C. Colpitts

Alex A. Compton

Jonas N. Conde

Kristen Lea Conn

Brian Cooke

Kevin M. Coombs

**Juliana Reis Cortines**

Sara Louise Cosby

Laurent Coscoy

Luciana Jesus Costa

Christopher Cottrell

Andrea L. Cox

Cristina Crava

Jeremy Chase Crawford

Lindsey B. Crawford

Thibaut Crépin

Robert W. Cross

Sheng Cui

Zongqiang Cui

Herrera da Silva

Bernadeta Dadonaite

Robert Daniels

Saumitra Das

April Dawn Davis

Patricia M. Day

Zeger Debyser

Kieran Dee

Cornelis de Haan

Leen Delang

Henri-Jacques Delecluse

Bernard Delmas

Zihni Demirbag

Maluquer de Motes

Kai Deng

Qiang Deng

Tao Deng

Wen Deng

John J. Dennehy

Rubelisa de Oliveira

Marc Desforges

William de Souza

Rik de Swart

Susan Detmer

**Robert de Vries**

Emmie de Wit

Ahmed Diab

Michael S. Diamond

Diego G. Diel

Ronald Dijkmann

Johannes Dijkstra

Chan Ding

Ron Diskin

Senad Divanovic

Richard D. Dix

Thomas Dobner

Ulrich Dobrindt

Terje Dokland

Patrick T. Dolan

Changzhi Dong

Florian Douam

Nir Drayman

Sarah Dremel

Ingo Drexler

Jan Felix Drexler

John Driver

Yuchun Du

Purnima Dubey

Rebecca M. DuBois

Mariette F. Ducatez

Janine A. Duckworth

Ralf Duerr

Nisha K. Duggal

Roy Duncan

David D. Dunigan

Paul T. Edlefsen

Edward H. Egelman

Catherine Eichwald

Shirit Einav

Nels C. Elde

Gillian Elliott

Samantha Emma Ellis

Matthew Elrick

Othmar Engelhardt

Armin Ensser

Andrea K. Erickson

Anna Erickson

Lucie Etienne

Mohamed Faisal

Baochao Fan

Qing Fan

Wenchun Fan

Yuding Fan

Tibor Farkas

Rubaiyea Farrukee

Heinz Feldmann

Carl Feng

Li Feng

Wen-hai Feng

Xiuli Feng

**Zongdi Feng**

Maureen C. Ferran

Gianmarco Ferrara

Helena Lage Ferreira

Lucas M. Ferreri

Claudia V. Filomatori

Andrew Firth

Nicole Fischer

Adam T. Fishburn

Meghan Flagg

Louis Flamand

Ervin Fodor

Beatriz Fontoura

Suan-Sin Foo

Adriana Forero

Donald N. Forthal

Annette Fox

Ellen Flescher Foxman

Cornel Fraefel

Oliver I. Fregoso

Yuejun Fu

Marc Fuchs

Katsuhiko Fukai

Sarah Gallois-Montbrun

Llilianne Ganges

Chengjiang Gao

Fei Gao

Feng Gao

Qinshan Gao

Mauricio Garcia

Matthew Gartner

Laila Gasmi

Ron Geller

Chao Geng

Emmanuelle Genoyer

Robert J. Gifford

Robert Gilbertson

Elena E. Giorgi

Nandan S. Gokhale

Daniel H. Goldhill

Benjamin Goldman-Israelow

Wenjie Gong

Marco Túlio Gontijo

Carlos F. Gonzalez

Daniel Gonzalez-Dunia

Matthias Gotte

Leah Victoria Goulding

Adeline Goulet

Reingard Grabherr

Lisa Gralinski

Todd J. Green

Alex David Greenwood

Keith Grehan

Jennifer T. Grier

Anthony Griffiths

Dirk Grimm

Martin H. Groschup

William Groutas

Ian J. Groves

Jingmin Gu

Pablo Guardado-Calvo

Severin Gudima

Suryaram Gummuluru

Changjun Guo

Chunhe Guo

Guijie Guo

Hongyan Guo

Longjun Guo

Ravindra Kumar Gupta

Jurgen Haas

Jennifer Habel

Susan Hafenstein

Bumsuk Hahm

Peter Halfman

Roy A. Hall

Dustin Hancks

Grant Hansman

Walter Harrington

Audray K. Harris

Eva Harris

Mark Harris

Robert L. Harrison

Stephen C. Harrison

Amy L. Hartman

Graham F. Hatfull

David Hawman

Biao He

Guimei He

Hongbin He

Hongxuan He

Brook E. Heaton

Nagendra R. Hegde

Andrew J. Henderson

Rory Henderson

Christiane Herden

Eva Herker

Meghan Hermance

Jesus Hernandez

Luis Hernández-Pelegrín

Elisabeth A. Herniou

Bobby Brooke Herrera

Joanne Hewitt

Kyoko Hida

Hiroyuki Hikida

Deborah Hinton

Catarina E. Hioe

Alec J. Hirsch

Ryan Hisner

Jody Hobson-Peters

Thomas Hoenen

Xupeng Hong

Jay Hooper

Stacy M. Horner

Marc Horwitz

Myra T. Hosmillo

Gaopeng Hou

James Hoxie

Peter Hraber

Shie-Liang Hsieh

Jiao Hu

Yong Huang

Mathieu Hubert

Brydie Huckestein

Karthik Hullahalli

Adam Joseph Hume

Yuqi Huo

Nick Hurlburt

Edward Hutchinson

Jennifer Lynn Hyde

Alexander P. Hynes

Michael F. Hynes

Joseph M. Hyser

Mathieu Iampietro

Terumasa Ikeda

Tetsuro Ikegami

Jean-Luc Imler

Michael J. Imperiale

İkbal Agah İnce

Nerea Irigoyen

Stuart Isaacs

Olaf Isken

Masanori Isogawa

Michael Ison

Naoto Ito

Tijana Ivanovic

Hidetomo Iwano

Luke R. Iwanowicz

Zsuzsanna Izsvák

Robert Jackson

Terry Jackson

William Jacobs Jr.

Steven Jacobson

Rohit K. Jangra

Babal Kant Jha

Kuntong Jia

Dawei Jiang

Guochun Jiang

Ping Jiang

Yousheng Jiang

Zhongyi Jiang

Yuefei Jin

Bryan A. Johnson

Cydney N. Johnson

Karyn N. Johnson

Kenneth Johnson

Silas F. Johnson

Welkin E. Johnson

Clare Jolly

Melissa K. Jones

Terry C. Jones

Anan Jongkaewwattana

Colleen B. Jonsson

Joyce Jose

Lok Joshi

Michael Gordon Joyce

Sandra Junglen

Jennifer Ann Juno

Lars Kaderali

Denis E. Kainov

Naoko Kajitani

Spyros A. Kalams

Tatsuo Kanda

Sang-Moo Kang

**Sherin Kannoly**

Amit Kapoor

Jonathan Karn

Rachel A. Katzenellenbogen

Benedikt Kaufer

Takahiro Kawagishi

**Yoshihiro Kawaoka**

Satoshi Kawato

Helenie Kefalakes

Brian Kelch

Alyson A. Kelvin

Reza Khayat

Arthur S. Kim

Geon-woo Kim

Won-Il Kim

Benjamin King

Linda King

Renee E. King

Anja Kipar

Thomas Klose

Barbara G. Klupp

Dorota Kmiec

Oren Kobiler

David M. Koelle

Cassandra Koh

Jacob Kohlmeier

Keiichiro Koiwai

Ryuhei Kokusho

Andrey B. Komissarov

Hidehiro Kondo

Renate König

Richard Kormelink

Emek Kose

Anna Koslova

Mario Köster

Marios Koutsakos

Philip Krause

Veronika Krchlíková

Vasiliya Kril

Ersheng Kuang

Anil Kumar

Deepak Kumar

Alexandra Kupke

Jesse J. Kwiek

Constantinos Kyriakis

Bernard La Scola

Jason T. Ladner

Martin Laggin

Michael Lagunoff

Benjamin Lamp

Yungang Lan

Nathaniel Landau

Jennifer Landolfi

Johannes Langedijk

Dennis Lapuente

Georg Lauer

Kathlyn Laval

Mansun Law

Sean E. Lawler

Élcio Leal

Chung-Young Lee

Hyunwook Lee

Quentin Le Hingrat

Jian Lei

Petr G. Leiman

Valerie Le Sage

Michael Letko

Jiann-Horng Leu

Corri B. Levine

Mary K. Lewinski

**Bin Li**

Chaozheng Li

Chengcheng Li

Chengjun Li

Fangfang Li

Guoxin Li

Hongmin Li

Jida Li

Junhua Li

Lian-Feng Li

Lin Li

Mengfeng Li

Pei Li

Qi Ming Li

Shitao Li

Xiangdong Li

Yanhua Li

Yanpeng Li

Yuhuan Li

Zhaofei Li

Zhaolong Li

Chen Liang

Guoxin Liang

Qiming Liang

Yuying Liang

Min Liao

Jean K. Lim

Wenyu Lin

Lisa C. Lindesmith

William G. Lindsley

Daniel Lingwood

Hai-Peng Liu

Lei Liu

Lu Liu

Mafeng Liu

Quan Liu

Shengwang Liu

Wanhong Liu

Wei Liu

**Xing Liu**

Yao Liu

Yue Liu

Gang Long

Ana M. Lopes

Carolina B. Lopez

Alessio Lorusso

Gildas Loussouarn

Emma Kate Loveday

Jonathan Lovell

Crystal L. Loving

Arianne Lowe

Rosa Lozano-Duran

Maolin Lu

Rui Lu

Xiao-Hong Lu

Zhiqiang Lu

Martin Ludlow

Stephan Ludwig

Valeria Lulla

David Lunn

Dahai Luo

Joachim Lupberger

Huibin Lv

XuSheng Ma

Zhe Ma

Robin M. MacDiarmid

Todd MacFarlan

Mohamed Mahdi

Tokameh Mahmoudi

Andrea Maisner

Marian Major

Rama K. Mallampalli

Stanley Maloy

Mike Mandell

Joseph L. Mankowski

Jaclyn K. Mann

Mark Manzano

Peter Alan Maple

Florence Margottin-Goguet

Darren P. Martin

Verónica Martín

Luis Martinez-Gil

Mathias Martins

Mauricio A. Martins

Laura Martin-Sancho

Shin-Yi Lee Marzano

Enric M. Mateu

Keita Matsuno

Eiko Matsuo

Karen L. Maxwell

Jens Mayer

Richard Mayne

Alison McBride

Laura-Isobel McCall

Kevin McCarthy

Anita K. McElroy

Andrew T. McGuire

Dorian McIlroy

Gerald Michael McInerney

Jane A. McKeating

Alan McLachlan

Jason S. McLellan

Andrew McMichael

Christopher McMillan

Cynthia M. McMillen

Nathan Meade

Kavi P. Mehta

Jeffery L. Meier

Gregory B. Melikyan

**Vineet D. Menachery**

Luis Menendez-Arias

Bo Meng

Songdong Meng

Andres Merits

Robert C. Mettelman

Shuo Miao

**Eleftherios Michailidis**

Lefteris Michailidis

Dieter Mielke

Pascal Miesen

Barry Milavetz

William E. Miller

Ali Mirazimi

Chad Mire

Hiroaki Mitsuya

Mahesh Mohan

Rebekah L. Mokry

Arindam Mondal

Cary Moody

Michael A. Moody

Nathaniel J. Moorman

Raszl Simone Moraes

Hector R. Morbidoni

Christopher Mores

Yasuko Mori

Kohji Moriishi

Juliet Morrison

Walter N. Moss

Hugo Mouquet

Sara Moutailler

Joseph Mudd

Frauke Muecksch

Barbara Müller

Janis A. Müller

Shirin Munir

Vincent J. Munster

Christian Münz

Takayuki Murata

Denys Muzyka

Jean Nachega

Lieve M. Naesens

Mahmoud M. Naguib

Venugopal Nair

H.J. Nauwynck

Rayhane Nchioua

Vashi Negi

Frank Neipel

Ujjwal Neogi

Mihai Netea

Benjamin W. Neuman

Michael M. Nevels

Ben Newman

Johan Neyts

Christiane Krystelle Nganou-Makamdop

José L. Nieva

Peter Nilsson

Benjamin E. Nilsson-Payant

Tatsuya Nishi

Kazuo Nishigaki

Asami Nishimori

Zheng Niu

Christopher C. Norbury

Darren Obbard

David H. O'Connor

Una O'Doherty

Anthony J. O’Donoghue

Hiroyuki Ogata

Michael Oglesbee

Thomas H. Oguin

Molly Ohainle

Shinya Ohta

Tomoichiro Oka

Hugo Oliveira

Victoria A. Olsen

Yaw Shin Ooi

Claire E. Otero Cristina Risco Ortiz

Gabriel Ozorowski

Megha Padi

Mark Painter

Gustavo F. Palacios

Li-Long Pan

Pan Pan

Tao Pan

Xiaoyi Pan

Amanda R. Panfil

Dai-Wen Pang

Bernadett Papp

Bhavya Papudeshi

Catalina Pardo-Roa

Chorong Park

Jaekeun Park

Junbae Park

Alan L. Parker

Jerry M. Parks

Gabriel I. Parra

Ilaria Pascucci

David Pasdeloup

Matthew D. Pauly

Jean-Michel Pawlotsky

**Mark Peeples**

Scott Pegan

Philip Pellet

Chen Peng

Daxin Peng

**Rushika Perera**

Mariano Pérez-Filgueira

Juan Perilla

Andrew Perkosz

Karin E. Peterson

Marine J. Petit

Stephanie Pfaender

Christian K. Pfaller

Beatrix Pfanzagl

Sebastien Pfeffer

Andreas Pichlmair

Alla Piirsoo

Stanley A. Plotkin

Stefan Poehlmann

Joe Pogliano

Marie O. Pohl

Emma Poole

Leo L.M. Poon

Matteo Porotto

Robert David Possee

Jan Postberg

Ultan F. Power

Apurva T. Prabhakar

Vidya Mangala Prasad

Alexander M. Price

Juan Pu

Miguel Rodríguez Pulido

Joanna A. Pulit-Penaloza

Dohun Pyeon

Ping Qian

Liang Qiao

Wenjie Qiao

Aijian Qin

Cheng-Feng Qin

Yinghui Qin

Zhiqiang Qin

Enya Qing

Xusheng Qiu

Yang Qiu

Yinsheng Qiu

Victoria F. Queiroz

Gokul Raghunath

Masmudur M. Rahman

Franciszek Rakowski

Krzysztof Rakus

Holly Ramage

Sasirekha Ramani

Octavio Ramilo

Glenn Randall

Udaykumar Ranga

Deepashri Rao

Angela L. Rasmussen

Giselle Rasquinha

Anusyah Rathakrishnan

Sanjay M. Reddy

Alec James Redwood

Daniel B. Reeves

Matthew Reeves

Fulvio Reggiori

Natalia Rego

Guiping Ren

Yujie Ren

Peter Reuther

Ruy Ribeiro

Taissa Ricciardi-Jorge

Gautier Richard

Jonathan Richard

Alexsia Richards

Simone I. Richardson

James L. Riley

Agustina Rimondi

Espen Rimstad

Jacques Robert

Erle Robertson

Joana Rocha-Pereira

Rosemary Rochford

Rebecca J. Rockett

Nicole Rockey

Rodrigo A. Rodrigues

Eduardo Rodríguez-Román

Mohammed Rohaim

José M. Rojas

Richard J. Roller

Fabio Romerio

Lijun Rong

Jason W. Rosch

Mette Rosenkilde

Maria Rosenthal

Ted M. Ross

**Andrew Routh**

David John Rowlands

Polly Roy

Nicolas Ruggli

Ezequiel Ruiz-Mateos

Till Rumenapf

Eugene Ryabov

Ivan Sabol

Tomohiko Sadaoka

Ivan Sadowski

Xavier Saelens

Selena M. Sagan

Manish Sagar

Akatsuki Saito

Daniel J. Salamango

Maria-Carla Saleh

Shadi Salloum

Parimal Samir

Clare E. Sample

Sabri S. Sanabani

Carlos Sandoval-Jaime

Sumana Sanyal

Erica Ollmann Saphire

Stefan G. Sarafianos

Alan Sariol

Mamta Chawla Sarkar

Anjana Sasidharan

Mamata Savanagouder

Bevan Sawatsky

Troels K. Scheel

Joshua T. Schiffer

Janke Schinkel

Anthony P. Schmitt

Katharina S. Schmitz

Mirco Schmolke

William M. Schneider

Tony Schountz

Sabrina Schreiner

Jürgen Schwarze

Stephanie Seifert

Stefan Seitz

Ruth Serra-Moreno

Liang Shan

Pengcheng Shang

Amit Sharma

Vivek Sharma

Timothy P. Sheahan

Chenguang Shen

Guobo Shen

Wei Shen

Lee Sherry

Mang Shi

Pei-Yong Shi

Masaki Shintani

Ikuo Shoji

Masahiro Shuda

**Deepak Shukla**

Longlong Si

Alex Sigal

Peter Simmonds

Francesco Simonetti

Amy C. Sims

Pawan Kumar Singh

Christian Sinzger

Rebecca L. Skalsky

Mikael Skurnik

Svetoslav Nanev Slavov

Richard Sloan

Nicolas Sluis-Cremer

Elena Smertina

Claire Mary Smith

Darci Smith

Everett Clinton Smith

Thomas E. Smithgall

Redmond Smyth

Joost Snijder

Ray T.Y. So

Cecilia Söderberg-Nauclér

Kenneth Söderhäll

Donald Sodora

Deping Song

Konstantin M.J. Sparrer

Peter Speck

Roberto F. Speck

Juliet Spencer

Adam Mitchell Spivak

Chelsey C. Spriggs

Megan E. Spurgeon

Peter Staeheli

Robert V. Stahelin

Robin Stanley

Richard James Stanton

Jack T. Stapleton

Tyler Starr

Gabriel J. Starrett

Spyridon Stavrou

Peter Stern

Caitlin Stoddard

Richard Strasser

Eva-Maria Strauch

Klaus Strebel

Benedikt Strunz

**Jianguo Su**

Ying-Hsiu Su

**Sundharraman Subramanian**

Andreas Suhrbier

Ampa Suksatu

Rebecca P. Sumner

Dongbo Sun

Yingjie Sun

Yuan Sun

M. Suresh

Mehul S. Suthar

Danica M. Sutherland

Masataka Suzuki

Luc Swevers

Taha Y. Taha

Christine Tait-Burkard

Kazuo Takayama

Mutsuyo Takayama-Ito

Tetsuo Takehara

Masaharu Takemura

Tomokazu Tamura

Gene S. Tan

Wenjie Tan

**Liudi Tang**

Shao-Jun Tang

Yan-Dong Tang

Pan Tao

Caroline Tapparel

Rachael Eugenie Tarlinton

Kishana Taylor

Matthew P. Taylor

Aartjan te Velthuis

Somporn Techangamsuwan

Han Kang Tee

Jose G. Teodoro

Olivier Terrier

David A. Theilmann

Volker Thiel

Andrea K. Thoma-Kress

David L. Thomas

Julie A. Thomas

Chih-Feng Tien

Ian Tietjen

Natasha Louise Tilston-Lunel

Anne L. Timmerman

Nicole D. Tischler

Michal Toborek

Costin Tomescu

Keizo Tomonaga

Montserrat Torremorell

Domenico Tortorella

Greg Towers

Sascha Trapp

Martha Triantafilou

Mirko Trilling

Jakob Trimpert

**Ralph A. Tripp**

Athe M.N. Tsibris

Kyoko Tsukiyama-Kohara

Jiagang Tu

Thomas Tu

Jessica Mitchell Tucker

Massimo Turina

Anne-Marie Turner

Kenneth L. Tyler

Alexandar Tzankov

Chitra Upadhyay

Jason W. Upton

Eeva Johanna Vainio

Vikram N. Vakharia

Jolien Van Cleemput

Neeltje van Doremalen

James Van Etten

Martijn van Hemert

Linda van Oosten

Sarah Van Tol

Nikos Vasilakis

Ravindra P. Veeranna

Michael Veit

Hana Veler

Elisa Vicenzi

Jefferson Russo Victor

Gonzalo Vigara-Astillero

Marco Vignuzzi

Maija Vihinen-Ranta

Damien Vitour

Romain Volmer

Dmitriy Volokhov

Asisa Volz

Jens von Einem

Mark von Itzstein

Hiep L. Vu

Stephen N. Waggoner

Jennillee Wallace

Derek Walsh

Xiu-Feng Wan

Aibing Wang

Bin Wang

Bo Wang

Gao-Xue Wang

Jian-Hua Wang

Jin-Xing Wang

Jiufeng Wang

**Jun Wang**

Lin-Fa Wang

Qiuhong Wang

Ran Wang

Shaowen Wang

Taia T. Wang

Tian Wang

Xiangxi Wang

Xian-Wei Wang

Xiao-Jia Wang

Xiao-Wei Wang

Xinglong Wang

Yiping Wang

Yue Wang

ZhaoFei Wang

**Brian M. Ward**

Cody J. Warren

Brian R. Wasik

Koichi Watashi

Christopher M. Waters

Michael Way

James Weger-Lucarelli

Jin Wei

Jingguang Wei

Wei Wei

Zhanyong Wei

Jason B. Weinberg

Carol D. Weiss

Joerg Thomas Wennmann

Theodore C. White

Matthew S. Wiebe

Thomas E. Wileman

Wessel Willemsen

Mark C. Williams

Zachary H. Williams

Justin E. Wilson

Pamela Tinmoi Wong

Sook-San Wong

Stefan Worgall

Antoni Wrobel

Jianjun Wu

**Kai Wu**

Shuwen Wu

Wenbi Wu

Zhiwei Wu

Emanuel Wyler

Dehui Xi

Yuchen Xia

Shaobo Xiao

Shuqi Xiao

Weidong Xiao

Hang Xie

Liangqi Xie

Xiaoli Xiong

Xingang Xu

Huan Yan

Jian Yang

Kai Yang

Kankan Yang

Jian Ye

Jing Ye

Xin Yin

Dongwan Yoo

Kyoung-Jin Yoon

Ian A. York

Jacob Yount

Meisam Yousefi

Hai Yu

Naitong Yu

Qigui Yu

Junfa Yuan

Meijin Yuan

Weiming Yuan

Xuefeng Yuan

Kwok-Yung Yuen

Hovakim Zakaryan

Cong Zeng

Francisco Murilo Zerbini

Shao-Lun Zhai

Beibei Zhang

Bo Zhang

Jun Zhang

Leiliang Zhang

Li Zhang

Lizhou Zhang

Qi Zhang

Qiwei Zhang

Wei Zhang

Wenyan Zhang

Xiaobo Zhang

Xiaonan Zhang

Yi-Bing Zhang

Zhenyu Zhang

Haiqing Zhao

Hongda Zhao

Ling Zhao

Mengmeng Zhao

Qin Zhao

Tiejun Zhao

Wenran Zhao

Xiaomin Zhao

Xuesen Zhao

Haixue Zheng

Jian Zheng

Kai Zheng

Shijun J. Zheng

Zhenhua Zheng

Bin Zhou

Hongbo Zhou

Tong Zhou

Xi Zhou

Xia Zhou

Xueping Zhou

Yan Zhou

Yu Zhou

Bin Zhu

Huanzhang Zhu

Jian Zhu

Qiyun Zhu

Yibin Zhu

Joseph Ziegelbauer

Adam Zlotnick

Sergei Zolotukhin

